# High-resolution definition of the *Vibrio cholerae* essential gene set with hidden Markov model–based analyses of transposon-insertion sequencing data

**DOI:** 10.1093/nar/gkt654

**Published:** 2013-07-30

**Authors:** Michael C. Chao, Justin R. Pritchard, Yanjia J. Zhang, Eric J. Rubin, Jonathan Livny, Brigid M. Davis, Matthew K. Waldor

**Affiliations:** ^1^Division of Infectious Disease, Brigham & Women’s Hospital, Boston, MA 02115, USA, ^2^Department of Microbiology and Immunobiology, Harvard Medical School, Boston, MA 02115, USA, ^3^Howard Hughes Medical Institute, Boston, MA 02115, USA, ^4^Department of Immunology and Infectious Diseases, Harvard School of Public Health, Boston, MA 02115, USA and ^5^Genome Sequencing and Analysis Program, Broad Institute, Cambridge, MA 02142, USA

## Abstract

The coupling of high-density transposon mutagenesis to high-throughput DNA sequencing (transposon-insertion sequencing) enables simultaneous and genome-wide assessment of the contributions of individual loci to bacterial growth and survival. We have refined analysis of transposon-insertion sequencing data by normalizing for the effect of DNA replication on sequencing output and using a hidden Markov model (HMM)-based filter to exploit heretofore unappreciated information inherent in all transposon-insertion sequencing data sets. The HMM can smooth variations in read abundance and thereby reduce the effects of read noise, as well as permit fine scale mapping that is independent of genomic annotation and enable classification of loci into several functional categories (e.g. essential, domain essential or ‘sick’). We generated a high-resolution map of genomic loci (encompassing both intra- and intergenic sequences) that are required or beneficial for *in vitro* growth of the cholera pathogen, *Vibrio cholerae.* This work uncovered new metabolic and physiologic requirements for *V. cholerae* survival, and by combining transposon-insertion sequencing and transcriptomic data sets, we also identified several novel noncoding RNA species that contribute to *V. cholerae* growth. Our findings suggest that HMM-based approaches will enhance extraction of biological meaning from transposon-insertion sequencing genomic data.

## INTRODUCTION

The coupling of high-density transposon mutagenesis with high-throughput DNA sequencing (referred to here as ‘transposon-insertion sequencing’) has given microbiologists a potent new tool—the capacity to simultaneously and comprehensively assess in a genome-wide fashion the contributions of individual loci to growth. There are many different methods for transposon-insertion sequencing, using different transposons and methods for library preparation {e.g. TnSeq, InSeq, TraDIS and HITS [reviewed in (1,2)]}. However, all transposon-insertion sequencing studies start with the construction of a high-density transposon library, which in principle can contain mutations disrupting every nonessential locus in the genome. Then, next-generation DNA sequencing is used to map transposon insertion sites en masse. Quantitative analyses of the frequency with which mutations in each genetic locus are detected enables discrimination between loci that are required or dispensable for growth under the conditions of interest. Mutations should be absent in essential loci and underrepresented in loci that promote, but are not required for, survival and proliferation.

Since its independent introduction in 2009 by four different groups (3–6), transposon-insertion sequencing approaches have been applied to study diverse bacteria under a variety of growth conditions [see (1,2)]. However, most past and current methods for analysis of transposon-insertion sequencing data do not take full advantage of the information stored within these large data sets. For example, even though these studies yield genome-wide information, most analyses only characterize annotated open reading frames (ORFs) and thus omit consideration of untranslated loci such as *cis*-acting sites and noncoding RNAs (ncRNAs) (1). Moreover, no transposon-insertion sequencing study reported has fully exploited the local context information inherent in high-density transposon libraries. In general, insertion mutants within the same genetic locus have a high probability of causing similar phenotypes. However, due to several technical and biological sources of noise, including library undersaturation, amplification bias/jackpots and stochastic variation between replicates, the number of reads for insertion mutants at adjacent sites can vary widely. Such noise can sometimes obscure biologically meaningful patterns within the data, especially when reads for each locus are averaged together.

In this work, we exploit a hidden Markov model (HMM) to improve previous analyses by lessening the impact of intrinsic variability (noise) in transposon-insertion sequencing data and generating more robust and consistent biological signals. HMMs have been widely used to find meaningful patterns in many bioinformatics contexts, including DNA alignment, protein fold prediction and motif searching (7), but not yet for analyses of transposon-insertion sequencing data. The HMM calculates the most likely sequence of nonobserved ‘hidden states’ given a series of observed ‘emissions’. Specifically, when adapted for mariner-based transposon-insertion sequencing data, the number of reads at each insertion site (emissions) enables discovery of the hidden identity of each TA site (i.e. whether the site is in an ‘essential’, ‘neutral’ or ‘sick’ locus). The HMM approach creates a statistical framework that incorporates local context into predictions, so that the hidden identity of each TA site is inferred by both its own reads and those from the previous TA site. This approach is relatively insensitive to read count noise that can confound previous analysis methods, and creates a genomic profile (i.e. all regions that are essential for growth should display a similar pattern of reads) that can be used to functionally separate genomic loci into different biological categories.

Here, we present a high-resolution transposon-insertion sequencing-based study of the contribution of all genetic loci to growth in rich medium for *Vibrio cholerae*, the Gram-negative etiologic agent of the diarrheal disease cholera. We categorized all genes and intergenic loci in the genome based on their contribution to the growth and survival of the organism *in vitro,* using an HMM-based secondary analysis that enhanced our ability to discern essential gene signals from noise. Because transposon-insertion sequencing methods are constrained by sequencing capacity, we use ‘essential’ in this study to refer to loci that are absolutely necessary for survival as well as to loci whose interruption compromises growth to the point that they are not represented in the sequenced library. Comparison of the *V. cholerae* essential gene set to that of *Escherichia coli* identified differences between these related organisms in critical metabolic and physiologic processes. Moreover, the HMM allowed us to identify a new class of ‘sick’ genes whose inactivation impaired growth. We also identified *cis*-acting noncoding essential loci, including many putative 5′ untranslated regions (UTRs) of essential genes. Finally, by comparing our data set with transcriptome data (8), we identified candidate *trans* acting ncRNA species that are required for optimal *V. cholerae* growth. Collectively, our study provides a highly refined curation of the essential *V. cholerae* gene set, and illustrates the utility that HMM-based analyses can bring to transposon-insertion sequencing data sets.

## MATERIALS AND METHODS

### Strains, media and culture conditions

All strains were grown on LB Miller (1% NaCl) unless otherwise noted. Antibiotic concentrations used were 200 µg/ml streptomycin (Sm), 50 µg/ml kanamycin (Km) and 100 µg/ml ampicillin (Amp). Wild-type *V. cholerae* C6706 and *Escherichia coli* SM10 lambda *pir* carrying the Himar1 suicide transposon vector pSC189 (9) were grown at 37°C in LB + Sm and LB + Amp, respectively. Individual transposon mutants from the ordered *V. cholerae* transposon library (10) were propagated in LB + Sm + Km at 37°C, overnight, unless otherwise stated. Where indicated, transposon mutant strains were grown in M9 media + 0.2% glucose + Sm + Km. Riboflavin (Sigma) was supplemented as indicated.

### Transposon mutant library construction

Three independent transposon libraries were created in *V. cholerae* C6706 through conjugation. Briefly, 1.6 ml of an overnight culture of SM10 lambda *pir E. coli* carrying a suicide transposon vector, pSC189 (9), was mixed with 1.6 ml of an overnight culture of *V. cholerae* C6706. Cells were pelleted, media removed, washed once with LB and then resuspended in 800 µl LB. Hundred microliters aliquots of the resuspended cells were spotted onto 0.45 µm HA filters (Millipore) on an LB agar plate. The mating plate was incubated at 37°C for 2 h, then all the filters were put into a single tube with 7.5 ml LB and the cells resuspended by vortexing. The resuspended cells were then equally plated onto three 245 × 245 mm^2^ (Corning) LB + Sm + Km agar plates and colonies grown at 30°C for 24 h.

Colonies on the library plates were scraped, resuspended in LB and then pooled in a final volume of ∼15 ml. A 3 ml aliquot of cells was used for genomic DNA extraction using the Wizard Genomic DNA extraction kit (Promega), using at least 10× volumes of all reagents for this lysis. At the final DNA precipitation step, isopropanol was added and the precipitated genomic DNA was spooled out of the isopropanol solution using a blunted glass Pasteur pipette. The DNA pellet was then successively washed in 70 and 100% ethanol, spooling between each wash into a new tube. After the final wash, excess ethanol was pressed out of the DNA with a pipette and then air dried at RT for 30 min. Dry DNA pellets were finally resuspended in 1.5–2.5 ml nuclease-free water at 65°C for at least 1 h, with frequent mixing.

### Transposon junction amplification, sequencing and mapping

In general, transposon junctions were prepared and amplified according to a previously published method (11). However, we digested our genomic DNA using Fragmentase (NEB) as opposed to mechanical shearing—specifically, 25 µg genomic DNA from each replicate library was treated with Fragmentase (five reactions worth of enzyme in a 250 µl solution for 1–1.25 h at 37°C). After fragmentation, DNA was end repaired (Quick Blunt kit, NEB), adaptors ligated (sequences in Supplementary Table S15) and PCR amplification conducted as previously described. The final PCR amplified products were gel purified on a 2% agarose gel to isolate 200–500 bp fragments, and subjected to qPCR using primers against the Illumina P5 and P7 hybridization sequence (Supplementary Table S15) to estimate input and ensure equal multiplexing in downstream sequencing. Equimolar DNA fragments from each library were combined and then sequenced on the MiSeq V2.0 platform for 65 cycles using a 50-cycle cartridge (Illumina).

Libraries 1–3 had the following number of total reads: 812 415; 1 857 563; 1 510 158. Reads were trimmed for transposon and adaptor sequences using CutAdapt (12), and then mapped to the *V. cholerae* N16961 genome with three allowable mismatches using the Bowtie aligner (13). Note that *V. cholerae* El Tor clinical isolates, like C6706 and N16961, only differ by ∼50–250 single nucleotide polymorphisms (SNPs) across the entire genome (14). Duplicated loci were not mapped and are thus absent in the study—these loci, as well as loci absent in *V. cholerae* C6706 compared with *V. cholerae* N16961 (personal communication) are listed in Supplementary Table S7. For libraries 1–3, the number of total mapped reads were 375 775, 588 918 and 494 757 (∼30–45% mapping efficiency), which hit 31, 33 and 35% of all TA dinucleotides in the genome, respectively. After mapping, the reads for each TA site were tallied, assigned to a known gene or an intergenic region and then visualized on DNAplotter or Artemis (15). Finally, each library that contained a similar percentage of TA sites hit was normalized to 375 000 total mapped reads and combined into a single data set covering ∼1.1 million reads in the genome (and insertions at 56% of all TA sites) using R (16).

### Positional correction for replication and hit distribution analysis

To normalize for chromosomal replication biases in read counts, chrI and chrII were divided into 10 kb nonoverlapping windows starting with windows centered around the origins. Using Microsoft Excel functions, the number of reads within each window (after removing insertion sites that have no reads to reduce overscaling due to essential loci) were normalized based on a factor calculated from the average read depth within the window divided by the average depth for the chromosome.

After normalization, the fraction of TA sites containing a transposon insertion was calculated for all genes. The genes were then sorted into bins based on their fraction of TA sites hit by the transposon, and this distribution was plotted. We visually estimated that the distribution of random insertions in neutral genes had a peak centered at 0.75. Counting the number of genes on the right half of this distribution enabled us to calculate the boundary on the left side of the peak that encompasses the same number of genes. Using this boundary, we used Excel to calculate the fraction (∼0.34) where the bottom 1% of neutral genes would be found. Five hundred two genes below this cutoff (the Hit Distribution or Hit Dist List) were underrepresented for transposon insertion and used to compare with other downstream analysis pipelines [i.e. the sliding window (SW) and HMM data sets].

### SW analysis

Mapped reads were then run with custom scripts as part of the SW pipeline; scripts (in Supplementary Methods) and example input files are found in Supplementary Materials (11). Briefly, for every TA-site count *X*, null distributions were generated from our data set by totaling the number of reads for *X* randomly sampled sites 10 000 times. Then, the number of reads was monitored at 10 bp intervals across the genome for overlapping 200 bp windows, and these numbers were compared with our null distribution. For each window, a *P*-value was calculated as the rank of the test mean divided by 10 000. The false discovery rate (FDR) of each window was calculated using the Benajmini–Hochberg test (17). Definition of significantly underrepresented windows in this data set (*P* < 0.01 and FDR < 0.05) required the presence of at least seven TA sites without reads. All the windows across a locus were then filtered with the following criteria: loci containing only significantly underrepresented windows (*P* < 0.01, FDR < 0.05) were assigned as essential, while loci containing only nonsignificant windows were assigned as neutral. Loci that contain at least one significant and nonsignificant window were assigned as domain essential. Loci that contained windows with too few TA sites to be capable of reaching statistical significance (less than seven TA sites) were unable to be evaluated. Intergenic loci were defined as any sequence that lies entirely between the end of a previous gene and the start of the current one. All intergenic regions are 5′ to their locus number on the forward strand [e.g. IG_VC0001 comprises nucleotides 1–324 (the bp before the coding sequence of VC0001)]. Analyses were performed using Python 2.7.3 with NumPy 1.7.1 and SciPy 0.12.0 add-on packages.

### Hidden Markov modeling and functional categorization of loci

To adapt the HMM framework to our mariner-based transposon-insertion sequencing data, we made the following analogy: hidden states are ‘essential’, ‘neutral’ and ‘sick’. Emissions are read counts at a given TA site. To adapt continuous read count data to a discrete modeling framework, we binned our data based on the distribution of read counts across all TA sites. The bin size was chosen to be the smallest number of bins that allowed us to separate true-positive sick genes (visualized in Artemis) from true-positive essential genes. For this analysis, the bin size was seven and all reads were discretized into each bin. Following discretization, we trained our HMM on blocks of SW calls across the entire genome—TA sites in underrepresented windows were assigned as essential, while TA sites in nonsignificant windows were assigned as neutral. To incorporate sick genes, we manually annotated our small set of 20 candidate sick genes (Supplementary Table S8) in the training set (these genes were chosen as they appeared to consistently have lower read depth compared with likely neutral loci). The initial estimates of the emission and transition probability matrices were derived using the Baum–Welch algorithm implemented in Matlab R2011b (Mathworks) (code in Supplementary Methods). Following the initial training, the sequence of hidden states was uncovered by implementing the Viterbi method. Furthermore, because exact training sets may produce biases, we iteratively refined the hidden states by using the sequence of the first iteration to rederive transition and emission matrices for the next iteration of the Viterbi parse. We did this until the emission and transition matrices were equivalent in subsequent iterations. Iteration is necessary to reduce the bias of a given training set. Different training sets can give different answers in the first implementation of the Viterbi algorithm, but in practice, iteration converges to the same set of predictions, even if the training sets vary.

After the HMM ran to convergence (i.e. the algorithm no longer changed assignments as it looped through the data), a set of secondary filters (Supplementary Methods) were used to functionally separate loci into the appropriate categories. Essential genes were defined as those whose central 80% coding sequence contained only TA sites classified by HMM as essential. Essential intergenic regions had only essential TA sites in the entire intergenic space. Neutral genes and intergenic regions were defined as regions containing only neutral TA sites. Regions not classified as essential or neutral loci were investigated as potential domain essential and sick regions. To be classed as domain essential, genes/loci had to contain both nonessential TA sites and a contiguous stretch of at least five essential TA sites, which covers 15–300 bp in 90% of all loci in the genome. Regions were classified as sick when they had a majority of ‘sick’ TA sites across the entire locus. Regions that did not meet criteria for domain essential or sick categories were placed into the neutral category. To calculate the confidence for each HMM assignment, we assessed the posterior probabilities (likelihood) for every TA site being categorized as essential, neutral or sick. The data were calculated using a modified version of the ‘hmmdecode’ function in Matlab (code is available in the Supplementary Methods), and the average probabilities for all loci are presented in Supplementary Table S14 (raw data are in the Supplementary Sample input file folder). The inputs for the ‘hmmdecode’ function were the transition and emission probability matrices and readcount emissions from the final iteration of the HMMconvergence script.

### Growth analysis of candidate sick and riboflavin synthesis mutants

A transposon mutant in candidate sick genes or in *ribE* (*vc2268*) was isolated from the ordered *V. cholerae* transposon library (10) and grown in LB + Sm + Km. Candidate sick genes were diluted 1:1000 in LB + Sm and grown at 37°C in a Bioscreen C optical density reader (Growth Curves USA) with OD600 measurements taken at 15-min intervals for 12 h. Additionally, candidate sick transposon mutants were diluted 1:1 with *lacZ^−^* wild-type *V. cholerae* and grown at 30°C, overnight. While the mutants from the ordered *V. cholerae* transposon library were constructed in a *lacZ^−^* background (10), the transposon used in that study carried a *lacZ* reporter gene, making these insertion mutants functionally *lacZ*+ and thus blue on X-gal media. Inoculum and overnight cultures were plated onto LB + Sm200 + 60 µg/ml Xgal and the resultant mutant (blue) and wild-type (white) colonies were counted. Competitive indices were calculated as the ratios of mutant and wild-type colony-forming units (CFUs).

#### ribE

Tn cells were grown from the ordered transposon library, pelleted, washed in phosphate buffered saline and streaked out onto agar plates either containing riboflavin [LB + Sm + Km (LB)], or M9 + glucose + Sm + Km + 5 µM riboflavin (M9 + B2) or without [M9 + glucose + Sm + Km (M9)]. Cells were grown for 48 h at 37°C to visualize colonies. Additionally, washed cells were also diluted 1:1000 in LB, M9 or M9 + B2 as above in the Bioscreen C and OD600 measurements taken at 15-min intervals for 24 h.

### Identification of ncRNAs important for optimal growth *in vitro*

Annotated known ncRNA species (18), putative transcriptional units identified through comparative genomics-based searches (19) and small intergenic RNAs found through analyses of RNA-Seq data sets (8) were overlaid on the SW results. Then, the *P*-values of all overlapping windows that fit within each ncRNA were averaged together, and those ncRNAs containing a window(s) statistically underrepresented for reads (*P* < 0.05) were identified as candidate species required for optimal growth.

## RESULTS AND DISCUSSION

### Generation of a high-density transposon library in *V. cholerae*

We used the well-characterized Himar1 mariner-based transposon vector pSC189 (9) to create a high-density transposon insertion library in *V. cholerae.* Himar1 inserts at TA dinucleotides without additional sequence constraints (20,21), rendering it extremely useful for high-density mutagenesis in an organism such as *V. cholerae*, which has a T + A of 52%. The *V. cholerae* genome consists of two unequally sized chromosomes, chrI (∼3 Mb) and chrII (∼1.1 Mb) (22). Comparative analyses of the two chromosomes from *V. cholerae* N16961 (a sequenced seventh pandemic El Tor strain) revealed that chrI has 2.75-fold more TA dinucleotides than chrII (∼140 000 versus ∼50 000), a difference that mirrors their 2.76-fold difference in size. In addition, the distances between adjacent TA sites on the two chromosomes are extremely similar, both within annotated genes and in intergenic sequences (Supplementary Figure S1A). Thus, mariner transposition is likely to be even across the genome, and both chromosomes can be analyzed using the same pipeline.

**Figure 1.**
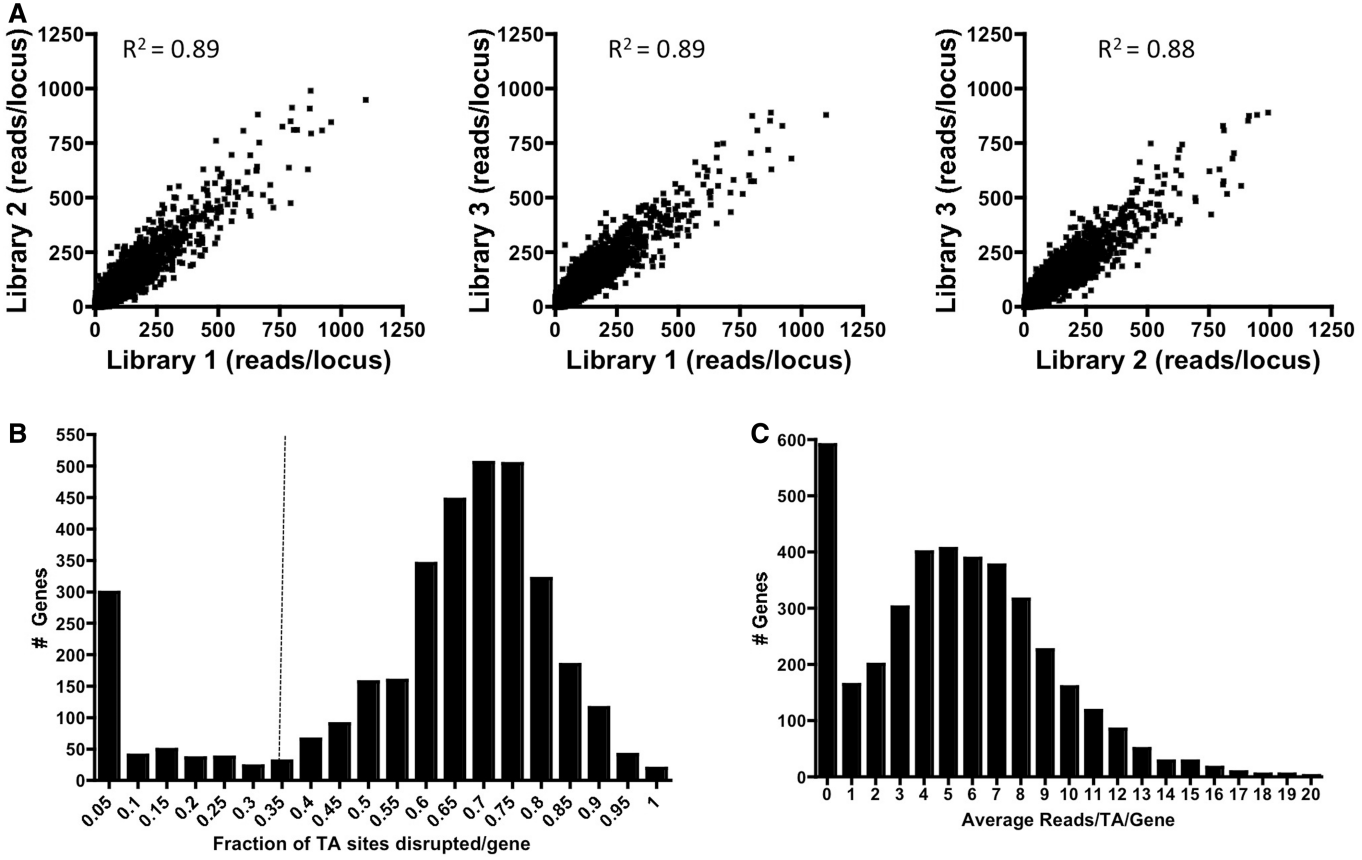
Reproducibility and distribution of Himar1 transposon insertions in *V. cholerae*. (**A**) The total reads from transposon insertions in each intergenic and genic region (∼6000 loci) were summed for each library. Pairwise comparisons of read abundance for each locus were plotted for the three libraries and used to determine the coefficient of determination (*R*^2^ value) between each pair. (**B**) The fraction of TA sites containing a transposon insertion was calculated for every gene in the pooled transposon-insertion sequencing data set, and the number of genes associated with each fraction is plotted. Using the distribution for presumed neutral genes (right peak), the 1% significance cutoff was calculated as 0.34 (dotted line). Genes to the left of this line have significantly fewer transposon insertions than expected for neutral insertion events (*P* < 0.01). (**C**) After positional correction, the total reads for all transposon insertions within each gene in the data set were averaged to the total TA sites in the gene. The number of genes associated with each average frequency is plotted.

We generated three independent transposon libraries, which together contained more than 600 000 transposon mutants (on average, ∼3-fold saturation of TA sites), in the seventh pandemic El Tor *V. cholerae* clinical isolate C6706 (10). The sites of Himar1 insertion in each library were determined by next-generation sequencing on the MiSeq platform and subsequently mapped onto the closely related *V. cholerae* N16961 genome. El Tor *V. cholerae* clinical isolates (such as N16961 and C6706) are thought to differ by only ∼50–250 SNPs across the genome (13), and we did not expect systematic difficulties in mapping C6706 reads to the N16961 reference genome. The number of insertions at each TA site was tallied, and then normalized such that each library contained equal numbers of mapped reads. Although the precise sites of insertion varied as expected among the libraries, when insertion sites were assessed for each annotated locus (∼6000 combined genic and intergenic loci), the composition of the three libraries appeared to be extremely similar. The Pearson correlation coefficient (*R*) between libraries was between 0.94 and 0.95, with coefficients of determination (*R*^2^ value) between 0.88 and 0.89 (Figure 1A). The high degree of concordance between the three biological replicates suggests that transposition occurred similarly in all three samples and subsequent amplification did not introduce distortion into our data set. Furthermore, this sequencing protocol has been previously found to accurately maintain the composition of each library (11). Therefore, we pooled our mapped reads together into a single high-density transposon insertion data set for downstream analysis.

### Distribution of transposon insertions

**Figure 2.**
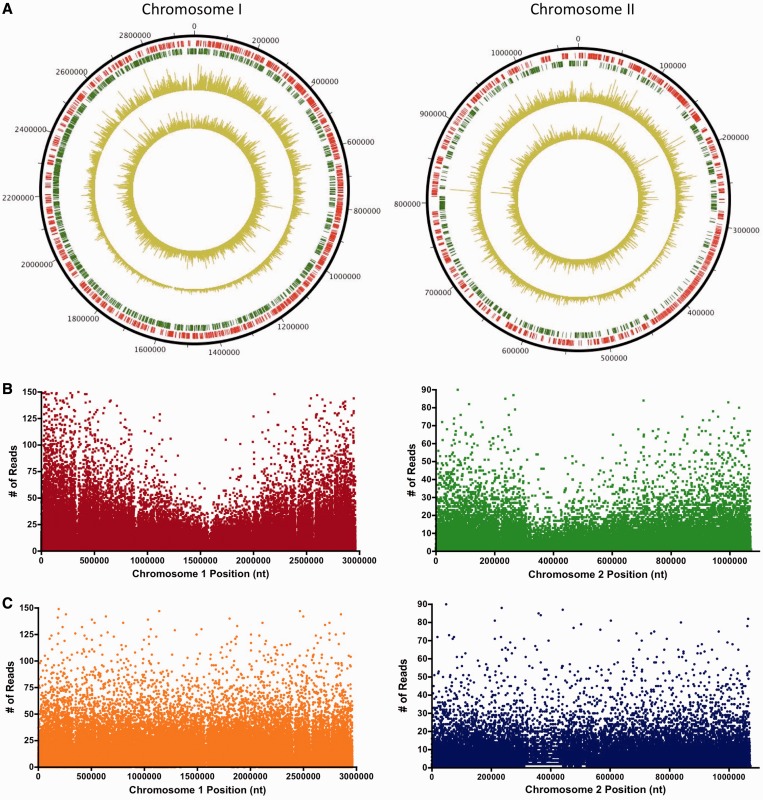
Normalization of transposon-insertion sequencing read abundance to correct for positional bias. (**A**) Reads were mapped onto both *V. cholerae* chromosomes; the amplitude of each peak represents the read abundance at a specific insertion site. Annotated genes encoded on the + strand are shown in red, while genes on the – strand are in green (outer track). Raw mapped reads are plotted on the outer gold track, while the position-normalized read abundance is shown on the inner gold track. (**B**) Raw mapped reads were plotted according to their genomic positions on chromosomes I and II. Origin proximal sequences are located at the ends of the graphs, while termini are located at or near the midpoints. Each spot represents a unique transposon insertion site. (**C**) Read abundance following normalization using a moving average smoothing method is plotted as a function of chromosomal position.

We plotted the position and read depth of each transposon insertion onto the two chromosomes to evaluate if there were any genomic ‘hotspots’ for transposition. Although transposon insertions mapped around both chromosomes (Figure 2A), there was a noticeable positional bias in read depth; for each chromosome, reads were most abundant at the replication origin region, and gradually decreased to a minimum level at or near the chromosome terminus (Figure 2A and B). This skew is likely due to the relative abundance of origin-proximal chromosomal regions that result from ongoing initiation of bidirectional DNA replication at these sites (23). An elevated number of chrI insertion sites, relative to chrII sites, were also observed (Figure 2B), consistent with the fact that chrII replication initiates once per cell cycle, whereas multiple chrI initiation events can occur (24,25). Thus, the positional skew in read depth appears to reflect the known replication-based skew in chromosome abundance.

While the positional bias is informative about *V. cholerae* chromosome replication dynamics, it interferes with comparison of the relative abundance of each transposon mutant in the library. To circumvent this issue, we normalized read depth for each chromosome with a position-dependent correction, which has been previously applied to transposon-insertion sequencing studies (26). Briefly, each chromosome was divided into 10 kb wide windows and the reads in each window were scaled to the average reads across the entire chromosome. The normalized data [Figure 2A (inner track), 2C] enabled direct comparison of transposon insertion frequency in all loci, regardless of genomic position.

The pooled library contained insertions in ∼56% of the TA dinucleotides in the genome. This translates to an average insertion of ∼15–16 transposons per locus in the library (Supplementary Figure S1B) and an average of 11 and 8 reads per TA position on chrI and chrII, respectively (Supplementary Figure S1C). TA sites lacking insertions could have either eluded transposition by chance or be located in regions that cannot tolerate insertions because they are essential for growth on LB plates. To distinguish between the two possibilities and estimate the true saturation of the library, we assessed the distribution of insertion frequencies per gene. As expected for subsaturating insertions within ‘neutral’ genes (i.e. genes whose inactivation has no impact on bacterial growth), we observed a large symmetric peak centered on genes with an insertion fraction of 0.70–0.75, suggesting these genes (and likely intergenic loci) on average underwent random transposon insertion at ∼75% of all available TA sites. Importantly, by estimating the area under the peak, we can set a 1 percentile cutoff for this curve at ∼34% of TA sites per gene disrupted (Figure 1B, dotted line). Notably, this cutoff yields 502 genes (hereafter referred to as the ‘Hit Dist’ gene set) with fewer transposon insertions than expected by chance alone (*P* < 0.01). Most of these ‘under-inserted’ genes (Supplementary Table S1) are likely loci that are either essential for survival or are required for optimal growth—i.e. transposon insertions in these genes render the cells ‘sick’ and thus less likely to be represented in the transposon library. However, additional analysis tools (discussed below) are required to distinguish between these possibilities.

### SW and hidden Markov analysis

Several different analytical approaches, each with their own benefits and drawbacks, have been used to identify genes required for growth using transposon-insertion sequencing data (3,11,27,28). Our data set, which has a high number of transposition sites but relatively few mapped reads per insertion in each locus on average (Figure 1C), permits evaluation of genomic insertion sites at high resolution, but requires an analysis methodology that does not depend on large differences in read depth between essential and neutral loci. The recent SW approach developed by Zhang *et al.* (11), in which the frequency of insertions in sequential overlapping windows is compared with a random null distribution based on the data set, allowed us to evaluate whether loci are underrepresented even at the relatively low read depth of our library. Furthermore, this approach enables analysis of variably sized genomic windows, allowing characterization of small loci, including intergenic regions and subdomains of ORFs, which other analyses do not explicitly address (28). Defining essential domains is valuable for evaluating the effect of truncation and point mutations within a locus, as well as allowing separate categorization of subregions within multi-domain genes for their contribution to growth. Alternative approaches that average reads across an entire locus may mask the presence of domains that cannot tolerate insertion.

In the SW pipeline, genomic regions in our data were randomly sampled to create a null distribution of expected reads for a given window size. Then, overlapping windows were scanned across the data and compared with the null distribution to assess whether a given window contained statistically fewer reads than expected by chance alone. In principle, the optimum SW would be small enough to resolve most protein domains and noncoding regions, while retaining a sufficient number of TA dinucleotides to reach statistical significance. We applied the SW algorithm (11) with minor modifications (see ‘Materials and Methods’ section) on our data using a 200 bp window. Then, all windows within a given locus were compiled, and a secondary filter used the *P*-values of each window to categorize all *V. cholerae* genes into one of three functional categories: (i) essential for growth, (ii) containing a domain essential for growth or (iii) neutral. With the SW analysis, 247 *V. cholerae* genes were classified as essential for growth (having only windows with significantly fewer reads in the core 80% coding sequence of the gene); 332 genes had putative essential domains (i.e. genes with both significantly underrepresented windows and neutral windows); 2759 genes were neutral for growth (did not contain underrepresented windows) and the significance of 412 genes was not able to be determined (Supplementary Table S1). Additionally, 136 genes in *V. cholerae* N16961 were either duplicated or absent in *V. cholerae* C6706 (Supplementary Table S7) and thus were not included in the analyses in this study.

**Figure 3.**
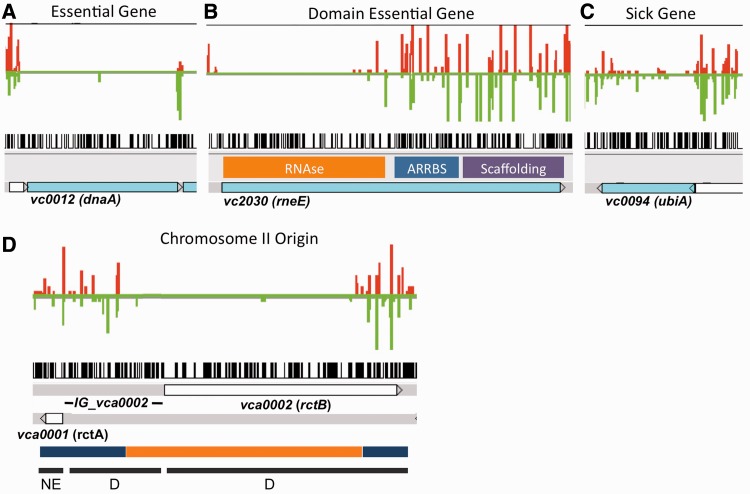
Examples of read abundance in essential, domain essential and sick genes and the chrII origin of replication (*oriCII*). Read abundance and sites of transposon insertions are shown for (**A**) essential, (**B**) domain essential, (**C**) sick loci and for (**D**) *oriCII*. Red bars represent the number of reads from insertions facing forward on the + strand and green bars reflect insertions on the complementary strand. Black bars represent all potential transposon insertion sites (TA dinucleotides) in the indicated regions. In (B), previously mapped *E. coli* RneE domains, including the RNase catalytic domain, the arginine-rich ribosomal binding site (ARBBS) and the scaffolding protein interaction domain were aligned to VC2030 and overlaid on the transposon-insertion sequencing data. In (D), transposon insertions are mapped across *oriCII*. The schematic spans the noncoding *vca0001* (*rctA*), the intergenic region *IG_vca0002* and *rctB* (*vca0002*), which encodes the initiator of chrII replication. Genetic analyses have revealed that the chrII origin includes regions dispensable (blue) and essential (orange) for DNA replication (29,30). Functional assignments from this study [NE = neutral locus, D = locus with an essential domain (subregion)] are shown at the bottom.

Although the SW approach is statistically robust, the secondary filtering of SW results appears overly stringent for assigning essential genes while being too permissive for domain essential candidates. For example, because essential ORFs are defined as genes with no underrepresented windows in the central 80% coding sequence in this approach, the known essential gene *dnaA* was erroneously assigned to the domain essential category due to a single window containing one read mapped within the gene (Figure 3A). True domain essential genes should contain long stretches of both essential and dispensable sequence (Figure 3B), but the SW filter does not assess this characteristic, which makes the approach susceptible to false-negative classifications of essential genes. Another limitation of the published SW approach is its inability to categorize genes that are not essential but are still required for optimal growth. Insertions in these ‘sick’ genes impair growth, and therefore, lead to fewer reads relative to neutral loci in the library (Figure 3C). In the SW pipeline, most *V. cholerae* genes that were ultimately validated as ‘sick’ (see below) were classified as nonessential because they contain reads across the gene (Supplementary Table S1).

To circumvent these limitations, we used a HMM to refine the SW predictions. HMM incorporates the local TA context of an insertion by acknowledging that the essentiality of any given site should take into consideration the number of mapped reads at an adjacent insertion site because transposon mutants in the same genomic region likely behave similarly. Specifically, the HMM uses a series of observed ‘emissions’ (the number of reads at each TA site) to calculate the probability of nonobserved hidden states (i.e. whether that site is within an ‘essential’, ‘neutral’ or ‘sick’ region). The HMM incorporates the read count at the previous TA site and its predicted state when evaluating the likelihood that the next insertion site belongs in the essential, neutral or sick category given its read count. However, the HMM requires a training set to estimate the likelihood of a TA site in an essential, neutral or sick region producing a specific read count. Thus, we trained the emission and hidden state probabilities for our HMM using the preliminary SW essentiality assignments at each TA site with their associated read counts, and then used the HMM to predict and refine the essentiality for every TA site in the genome. The logic and benefit of this approach is well illustrated by reexamining *dnaA* (Figure 3A). With the HMM-based analysis, the probability of having a stretch of essential TA dinucleotides followed by a single neutral TA site containing one read and then returning to another stretch of essential TA dinucleotides is highly unlikely; indeed, the HMM discounts the single read and assigns *dnaA* to the essential gene category (Supplementary Table S2).

In the same fashion, the HMM is able to use the local TA context to make calls that were not possible with the SW pipeline. The SW algorithm was unable to categorize 294 unique genes with fewer than seven TA dinucleotides because they possessed too few potential insertion sites to make a statistical determination of underrepresentation. However, the HMM could assign a functional categorization for these genes (Supplementary Table S6) by taking advantage of the information provided by adjacent loci. Because these genes did not benefit from having a preliminary essentiality assignment from the SW method, these gene calls may be less statistically robust (Supplementary Table S14) than genes containing more transposon insertions and are thus presented separate from the downstream analyses. Finally, the HMM was further trained with a small set of candidate genes (Supplementary Table S8) impaired in growth to detect regions that contain reduced transposon insertions and have lower read depth than truly neutral regions, i.e. regions corresponding to ‘sick’ genes. For example, in the case of *ubiA* (Figure 3C), which the SW algorithm classified as nonessential (Supplementary Table S1), the HMM recognized that the TA sites in this gene had fewer reads than in true neutral genes. Thus, the addition of HMM appears to generate more accurate assignments for essential genes than the SW pipeline alone and is also capable of categorizing small and sick genes.

### Comparisons between the HMM-based classification of essential genes and other analyses

**Figure 4.**
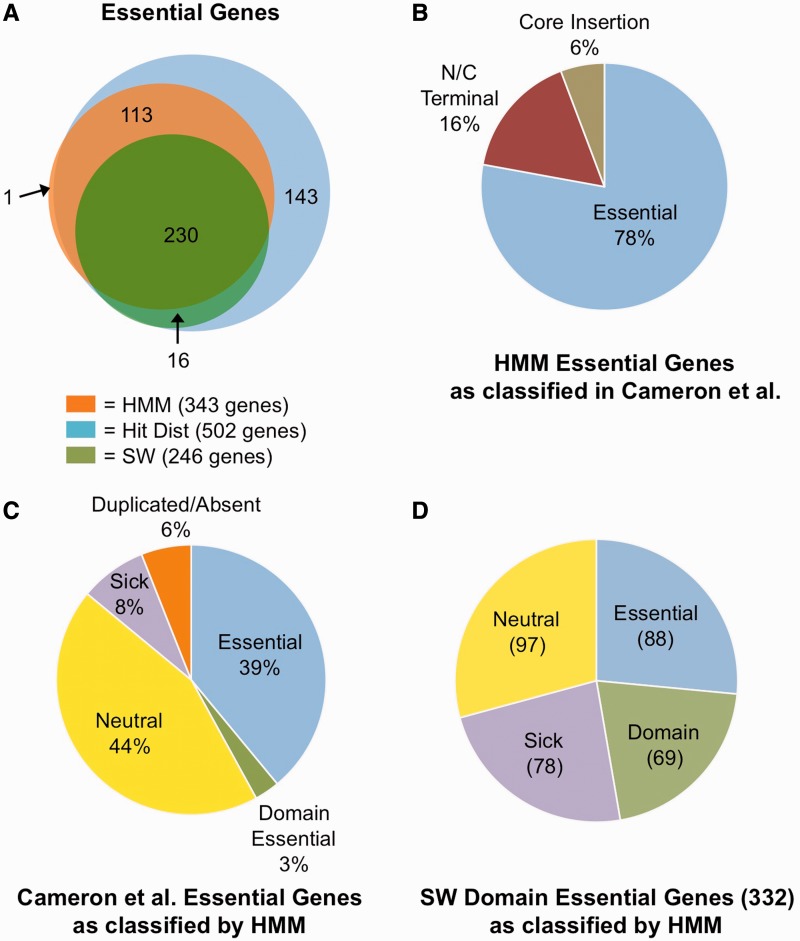
Comparative analyses of *V. cholerae* gene classifications. (**A**) Overlap of *V. cholerae* essential genes predicted using various analytical approaches. The overlap between essential genes predicted using HMM and SW analyses (SW) are shown, as well as genes on the Hit Dist list, which have a significantly low (*P* < 0.01) fraction of TA sites disrupted. (**B**) The 343 *V. cholerae* genes classified as essential based on HMM analyses were compared with the assignments of Cameron et al. Genes without representative transposon mutants in the ordered transposon library were marked as ‘Essential’, while genes that contained a transposon insertion were separated into two groups: those with an insertion within 10% of the ends of the genes (‘N/C terminal’), or those that had insertions within the central 80% of the gene’s coding sequence (‘Core insertion’). (**C**) HMM-based gene classifications are shown for the 789 putative essential genes defined by Cameron et al (10). The frequency of duplicated loci and genes that are not in the *V. cholerae* C6706 genome (duplicate/absent), which were not classified by HMM, are also shown. (**D**) HMM classification of the 332 genes identified as domain essential using SW analysis.

The HMM-based analysis of our data set yielded 343 essential, 91 domain essential, 128 sick and 2894 neutral genes with good confidence probabilities (Figure 4A and Supplementary Tables S2–S5, S14). It includes more (343 versus 246) than the number of *V. cholerae* genes classed as essential using the SW pipeline alone (Figure 4A), and given our hypothesis that the SW approach was more prone to false negatives, the HMM results are likely a more accurate list of true essential genes. This is supported by several independent analyses. First, nearly all (342 of 343) of the HMM essential genes overlap with the Hit Dist list, which identified 502 genes as having fewer transposon insertions than expected by chance alone (Supplementary Table S1, Figure 4A). Furthermore, 78% of the HMM essential genes were found to be essential (having no representative transposon mutant) in a previously defined ordered *V. cholerae* transposon library by Cameron et al. (10) (Figure 4B). Additionally, 16% of the HMM essential genes have transposon mutants in the ordered library in only the first or last 10% of the genes’ coding regions, consistent with the possibility [also suggested by Cameron et al. (10)] that disruptions at the termini of essential genes may sometimes not compromise gene function. In contrast, the Cameron library contains centrally located mutations (within the core 80% coding region) for only 6% of our essential gene candidates (Figure 4B). This discordance may represent false positives in the HMM assignments, though the confidence values for these genes appear good (Supplementary Table S14). Interestingly, our HMM essential genes list contained far fewer than the 789 essential genes identified by Cameron et al. Given their relatively small transposon library, they suggested that ∼350 of their essential candidates lacked transposon insertions owing to chance rather than being truly essential. Our data support this, indicating that 44% of these 789 genes have no apparent effect on *V. cholerae* growth (Figure 4C).

We also conducted comparative analyses of our predicted 343 essential genes against the 287 known essential genes in the related gamma-proteobacterium *E. coli* [as identified based on individual in-frame deletion studies (31)]. Homology searches revealed extensive concordance between the *E. coli* data set and our own (Supplementary Table S9)—homologs of 87% of the *E. coli* essential genes were also categorized as essential in *V. cholerae;* ∼2.5% of the *E. coli* genes did not have an apparent *V. cholerae* homolog, and a few of the remaining 10% of essential *E. coli* genes with *V. cholerae* homologs are in our sick gene list. In total, ∼90% of the *E. coli* essential genes that have *V. cholerae* homologs are either essential or promote optimal growth in *V. cholerae* (Supplementary Table S9). Collectively, there is good correspondence between our HMM results and two separate essential gene studies, suggesting that our HMM-based analysis is sufficiently sensitive to identify *V. cholerae’s* essential genes.

The HMM method also generated a noticeably smaller list of domain essential candidates compared with the SW approach (91 versus 332), which we might expect given that the SW filter categorizes domain essential as any gene with at least one essential and one nonessential window. This means that essential genes with even a single nonsignificantly underrepresented window, such as *dnaA* (Figure 3A), will be misclassified as domain essential. In fact, 88 (27%) of the genes on the SW domain essential list were classed as essential by the HMM (Figure 4D), and only 10 of these 88 have mutants that map to the central 80% of the gene in the Cameron ordered transposon library. In addition to underestimating essential genes, the SW domain essential list is also likely to include many genes that are in fact neutral. Because our library has only 75% saturation, it is likely that single underrepresented windows are present within neutral genes at a low but detectable frequency. Consistent with this idea, we found that 97 (29%) of the SW domain essential loci were classified as neutral by HMM, while 69 genes (21%) were shared between the two approaches (Figure 4D).

Although the set of HMM-assigned domain essential genes likely includes some false positives, our analysis is concordant with several previous analyses of individual loci. For example, HMM identified essential and dispensable regions within VC2030, which encodes ribonuclease E, an essential enzyme required for 5S rRNA processing (32) and mRNA turnover (33). The highly homologous *E. coli* RneE has three domains: (i) the ribonuclease catalytic domain; (ii) an arginine-rich RNA binding domain (ARRBS) and (iii) a C-terminal scaffolding region (34). Both the transposon insertions in VC2030 (Figure 3B) and genetic analyses of *rneE* in *E. coli* (34,35) indicate that the N-terminal catalytic domain is essential, whereas the ARRBS and scaffolding domains are dispensable for cell growth. Similarly, the HMM assigned *rctB*, which encodes the initiator of chrII replication, to the domain essential class. Transposon insertions were tolerated within the C-terminal 373 nucleotides of *rctB* (Figure 3D), consistent with genetic analyses that have revealed that deletions of up to 400 bp from the 3′ end of *rctB* do not abolish RctB’s ability to initiate chrII replication (29,36). Thus, our HMM facilitated the identification of essential genes that cannot tolerate insertions at any sites and reduced misclassification of neutral loci as domain essential in comparison with the SW method. However, further optimization of HMM predictions is possible to reduce false-positive assignments; for example, robust identification of domain essential loci can be favored by generation of more saturated transposon libraries, which have a lower chance of containing underrepresented windows by chance. Additionally, as domain essential candidates are validated, the likely size of an essential domain can be better estimated to allow more accurate filtering of genes with regions containing significantly fewer insertions.

### Identifying genes that contribute to optimum growth with the HMM

The HMM approach also facilitates identification of genes required for optimal growth because it permits detection of regions consistently represented by fewer reads than expected for growth-neutral genes. Such ‘sick’ genes are not readily distinguished from growth neutral loci using the SW pipeline, are missed by studies that treat insertions as binary events regardless of reads (28), and require an outgrowth step in other fitness-based studies (2). Our HMM results indicate that 128 genes are required for optimal growth of *V. cholerae* in LB (Supplementary Table S4). Interestingly, this list contains 72 genes that overlap the Hit Dist list (Supplementary Table S1), indicating that many of the sick genes not only had fewer templates (reads) in our library, but also had a low frequency of TA sites disrupted. In principle, the fraction of TA sites disrupted per gene should not vary between ‘sick’ and neutral loci. However, the relatively low read depth of our data (average of ∼10 reads per transposon mutant; Supplementary Figure S1C) likely results in some insertions at sick loci being undetected; in a library with greater read depth, we expect that the Hit Dist list would not contain sick genes.

**Figure 5.**
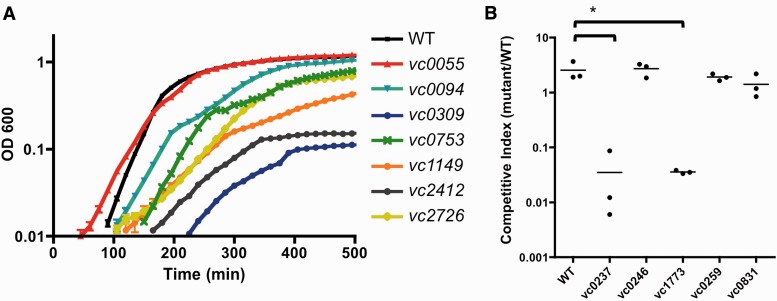
*In vitro* growth of mutants with transposon insertions in sick genes. (**A**) Culture density (OD 600) of wild-type *V. cholerae* and mutants with transposon insertions in ‘sick’ gene candidates is plotted as a function of time. (**B**) Competitive indices from *in vitro* competition assays between wild-type *V. cholerae* and mutants with insertions in candidate ‘sick’ genes are shown. Competitive indices were calculated as the ratios of mutant and wild-type CFUs after overnight co-culture in LB. *P < 0.001.

To validate the HMM assignments of sick genes, we compared the growth of insertion mutants in the candidate genes from the ordered *V. cholerae* transposon library (10) to the growth of wild-type cells. Mutants in sick genes that contained the lowest level of mapped reads (average 1–2 reads/insertion; Supplementary Table S4) exhibited a reduced growth rate *in vitro* for five of six genes tested (Figure 5A), confirming that the HMM analysis of transposon-insertion sequencing data can accurately identify mutations that result in growth defects. Insertion mutants in sick genes with higher average mapped reads also often exhibited some degree of growth impairment. For example, insertion mutants in *vc0309* and *vc0753,* which had average reads of 5.6 and 4.7, respectively, were severely attenuated in growth (Figure 5A). Likewise, *vc0237* and *vc1773* mutants (average of 3 and 5.4 reads per TA insertion) were less fit than wild-type cells in an *in vitro* competition experiment (Figure 5B). However, *vc0246*, *vc0259* and *vc0831* mutants (average reads per TA insertion of 5, 6.9 and 9.5, respectively) grew comparably with the wild-type strain (Figure 5B). Thus, the HMM calls of candidate sick genes appear to be more accurate with insertions containing lower average reads, while false-positive assignments are more likely for genes with higher average reads. Because we provided the HMM with a relatively small and uncharacterized training set of candidate sick genes, it should be possible to improve the accuracy of the HMM for detecting sick genes by training the algorithm with a larger set of biologically validated candidates.

### Differentially essential genes in *V. cholerae* and *E. coli*

It was initially surprising to find that 25 of the *E. coli* essential genes with *V. cholerae* homologs were neutral in our study (Supplementary Table S9). However, 11 of these genes appear to be duplicated in the *V. cholerae* genome and in most cases are likely to be functionally redundant (Table 1). For example, there are two nonessential paralogs of peptide deformylase (*vc0046* and *vca0150*), as well as aspartate semialdehyde dehydrogenase (*vc2107* and *vc2036*) in *V. cholerae*. Intriguingly, two additional paralogous pairs exhibited differential essentiality—of the tyrosyl tRNA synthetase paralogs *vc0465* and *vc0631,* only *vc0631* appears to be essential. Likewise, of the two genes encoding glycerol 3 phosphate acyltransferases (*vc0093* and *vc2024*), only *vc2024* is required for growth (Table 1). The nonessential paralogs may be pseudogenes, have diverged in function or be differentially expressed.

**Table 1.** Genes and functions of selected loci that are differentially essential in *V. cholerae* and *E. coli*

Essential homolog in *V. cholerae*	Neutral homolog in *V. cholerae*	Homolog in *E. coli*	Essential In *E. coli*?	Annotated function
*vc0631*	*vc0465*	*tyrS*	Yes	Tyrosyl tRNA synthetase
*vc2024*	*vc0093*	*plsB*	Yes	Glycerol 3 phosphate acyltransferase
	*vc0743*, *vca0693*	*secD*	Yes	General secretion pathway translocase

	*vc2269*, *vc1263*	*ribA*	Yes	GTP cylclohydrolase subunit II

	*vc2269*, *vca1060*	*ribB*	Yes	3,4 dihydroxy-2-butanone 4-phoshate synthase

	*vc2107*, *vc2036*	*asd*	Yes	Aspartate semialdehyde dehydrogenase

	*vc0058*, *vc0586*, *vca0274*	*can*	Yes	Carbonic anhydrase


	*vc0046*, *vca0150*	*def*	Yes	Peptide deformylase

	*vc2268*	*ribE*	Yes	Riboflavin synthase beta subunit
	*vc2270*	*ribC*	Yes	Riboflavin synthase alpha subunit
	*vc2271*	*ribD*	Yes	Riboflavin-specific deaminase
	*vc1714-vc1716*	*mukBEF*	Yes	Condensin subunits BEF
*vc2626*		*dam*	No	Adenine Methyltransferase
*vc2763-vc2767*		*atpCDGAH*	No	F1 ATP synthase (ε,β,γ,α,δ) subunits
*vc2768-vc2770*		*atpFEB*	No	F0 ATP synthase (B,C,A) subunits

After removing paralogous loci, there remain 14 nonessential genes in *V. cholerae* that are considered essential in *E. coli* (Supplementary Table S9). In a few instances, the differences between the two organisms may be explained by conditional essentiality. *ftsE* and *ftsX* are on the *E. coli* essential list, but *E. coli* strains with inactivating mutations in these genes can be grown in the presence of high salt (1% NaCl) (37). Most likely, *V. cholerae ftsE* and *ftsX* were assigned to the nonessential category because we isolated our insertion library on LB medium containing 1% NaCl. However, in other cases, differences in essentiality between *E. coli* and *V. cholerae* may reflect interesting differences in the two organisms’ physiologies (Table 1). For example, the five essential *E. coli* genes *ribABCDE* are required for synthesis of riboflavin (vitamin B2) [reviewed in (38)], but *ribCDE* homologs are dispensable in *V. cholerae* (*ribA* and *ribB* have paralogs in the genome). This observation suggests that *V. cholerae,* like *Lactobacillus* (39) and *Bacillus* species (40), may be able to use riboflavin present in the environment (e.g. in LB media), rather than relying solely on *de novo* synthesis. In support of this hypothesis, we found that a *ribE*::*Tn* (*vc2268::Tn*) strain does not grow on M9 media, but does grow on M9 supplemented with riboflavin (Supplementary Figure S2). Thus, although *V. cholerae* lacks recognizable riboflavin transporters, it is likely that this organism, unlike *E. coli*, encodes a riboflavin uptake mechanism that can substitute for *de novo* synthesis. In addition to *ribCDE*, homologs of *mukB, mukE* and *mukF*, which are required for chromosome compaction in *E. coli* (41), are also not essential in *V. cholerae* (Table 1). We readily detected *V. cholerae* with transposon insertions at these sites, and mutants are also present in the ordered transposon insertion library (10), raising the possibility that *V. cholerae* encodes machinery outside the *mukBEF* locus that can carry out genome compaction.

Of the 343 *V. cholerae* genes classified by HMM analysis as essential (at least for growth on LB), 56 can be disrupted or lack homologs in *E. coli* (Supplementary Table S2). Some of these genes may have been falsely classed as essential by HMM owing to their small size; however, the list is still likely to reflect true differences in bacterial processes between the two organisms, and does include previously described differentially essential loci, including *dam* (*vc2626*), *parA2* (*vca1114*) and *parB2* (*vca1115*) (Table 1) (42–44). The list also includes a number of antitoxin genes from putative toxin/antitoxin addiction loci, including *vca0360*, *vca0477*, *vca0486* and *vca0488* (Supplementary Table S2). Such genes are presumed to be essential when associated with active toxins, but the activity of most of *V. cholerae*’s numerous toxin/antitoxin pairs has not previously been assessed. Genes newly reported to be essential in *V. cholerae* include all seven genes of the F0/F1 ATP synthase locus (*vc2763-vc2770*). ATP synthase is essential in a variety of other bacteria (45–48), but not in *E. coli* (49), which can survive in the absence of the aerobic respiration machinery by fermentation (50). *Vibrio cholerae* may have metabolic requirements for fermentation that are not optimally available on LB. Thus, the comparison of essential genes between organisms can uncover distinct ecological and physiological requirements for each species.

### Predicted functions of the essential *V. cholerae* genes

**Figure 6.**
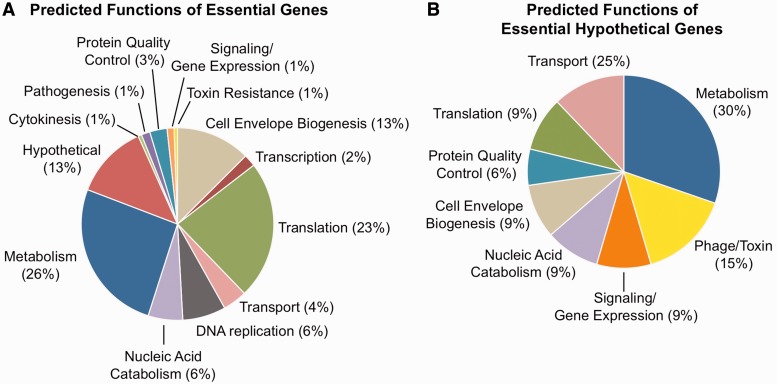
Essential gene processes in *V. cholerae.* (**A**) *V. cholerae* genes predicted to be essential based on HMM analysis were categorized by their GO terms into broader biological processes to highlight essential activities in *V. cholerae*. (**B**) Essential hypothetical proteins from (A) were subjected to homology searches and protein fold threading to evaluate if additional functional assignments could be made for these genes. These analyses allowed preliminary categorization of the biological roles for 32 of the 46 essential hypothetical proteins into general biological categories.

Most of the 343 *V. cholerae* essential genes we identified fall into four major categories [based on predicted gene ontology (GO) categorization in the annotated N16961 genome]: translation, metabolism, cell wall/envelope remodeling and hypothetical proteins (Figure 6A, Supplementary Table S10). The first three classes are expected because they represent conserved multicomponent pathways required for fundamental metabolic and structural functions in all bacteria, and such genes have predominated in studies of essential genes in other organisms (27,51,52). To better understand the role of the sequences initially annotated as hypothetical essential genes (53), which constitute 13% (45 genes) of essential genes, we performed sequence and protein structure homology searches. Notably, these analyses found medium to high confidence (e.g. PHYRE confidence values > 50%) functional predictions for 33 of these genes (Figure 6B, Supplementary Table S11). While a substantial number of these are predicted to be involved in metabolic and nucleic acid catabolism processes, there is also enrichment for phage and toxin-associated genes (15 versus 1% of total essential genes), such as the essential antitoxins described earlier (Supplementary Table S11). The prevalence of phage/toxin-related genes among the essential hypotheticals likely reflects the diversity and relatively understudied nature, yet centrality, of these horizontally acquired elements to bacterial growth and survival.

### Intergenic and noncoding regions required for *V. cholerae* growth

Most transposon-insertion sequencing studies reported to date have restricted their analyses to annotated open reading frames, with the exception of four studies (11,27,52,54). However, the SW and HMM methods enable a comprehensive analysis of the entire genome, and are therefore useful for defining unannotated regions that are important for growth, such as *cis* acting sites and ncRNAs. We used the HMM-based pipeline to functionally categorize every intergenic region within the *V. cholerae* genome (Supplementary Table S12 and S13), and identified more than 200 intergenic regions that appear to be essential or domain essential (Supplementary Table S12). The HMM-based assignments were consistent with experimental data. For example, they closely mirror previous genetic studies defining the essential sequences of the chrI and chrII origins of replication (oriCI and oriCII, respectively) (30,55). The precision of our transposon-insertion sequencing data is illustrated by the reads from the intergenic region that includes oriCII (*IG_vca0002*). The 5′ end of this intergenic sequence, which is known to be dispensable for replication, contains Himar1 insertions, whereas the 3′ sequence, which is known to be essential (30), contains no insertions (Figure 3D). In fact, the last insertion within this intergenic region occurred at position 753, the last TA dinucleotide before a DnaA box that is essential for replication. The HMM essential region also includes sequence upstream of *rctB*, which is essential for replication (30). Similarly, 41% of the essential or domain essential intergenic regions lie immediately upstream of predicted essential or domain essential genes, consistent with the expectation that promoters and some 5′ UTRs of essential genes should also be required for growth (Supplementary Table S12). In fact, two transcribed 5′ UTR regulatory sequences governing expression of essential ribosomal proteins [S15 (56) and Alpha RBS (57)] were defined as domain essential (Table 2), and other analyses have shown similar essential ribosomal leader sequences in other organisms (52).

**Table 2.** A selected list of intergenic regions significantly underrepresented for transposon insertion

Intergenic locus	Annotation	Category	Functional description
IG_1	Origin	E	Chromosome I origin
IG_vca0002	Origin	D	Chromosome II origin: the essential region is the last 380 nucleotides that contain the DnaA and RctB boxes
IG_vc2410	Rnase P	E	M1 ribozyme compoent of Rnase P, essential for tRNA and rRNA processing
IG_vc2574	Alpha_RBS	E	Ribosome binding site containing pseudoknot required for expression of 4 essential ribosome components
IG_vc0647	Rab_I_chrI_7	S	ncRNA discovered through transcriptomics; function unknown
IG_vc0431	C-CF	D	ncRNA discovered through transcriptomics; function unknown
IG_vc0143	B2	D	ncRNA discovered through transcriptomics; expression is significantly upregulated during mouse and rabbit infection
IG_vc0646	S15	D	Ribosome binding site containing pseudoknot required for expression of the essential ribosomal gene rpsO
IG_vc2473	RabM9_I_chrI_27	S	ncRNA discovered through transcriptomics; significantly upregulated during rabbit infection
IG_vca0933	M9_I5_chrII_36	D	ncRNA discovered through transcriptomics; significantly downregulated during rabbit infection, but upregulated in mice

Each intergenic region was classified as E = essential region; D = domain essential (contains a subdomain that is essential); S = ‘sick’ (nonessential, but required for optimal growth *in vitro*).

Underrepresented intergenic sites that are not associated with essential genes may represent trans-acting ncRNA loci required for optimal growth. While there are few known essential ncRNAs, the HMM algorithm correctly identified the RNase P ncRNA M1 as essential (Table 2). M1 is a ribozyme that forms the catalytic core of RNase P, an essential enzyme required for processing pre-tRNA and other RNA species (58,59). The HMM also classified as sick or domain essential several intergenic regions that encode *V. cholerae* ncRNAs [(8) and unpublished]. In subsequent analyses, we assessed the extent of correspondence between experimentally identified ncRNA coordinates and regions found via SW analysis to be underrepresented among insertion sites. This approach enabled identification of eight intergenic transcribed loci associated with significantly fewer sequencing reads (*P* < 0.05), which are likely to be required for optimal growth *in vitro* (Table 2). These ncRNAs encode the previously mentioned RNase P M1, S15 and Alpha RBS ncRNAs, as well as five novel ncRNA species whose functions remain uncharacterized. Interestingly, at least two, B2 and M9_15_chrII_36, have significantly different expression under *in vitro* and *in vivo* conditions [(8) and unpublished]. Additional combined analyses of transposon-insertion sequencing and transcriptomic data from cells grown under diverse environmental conditions should be a powerful approach for identifying regulatory ncRNAs that mediate cell survival under stress conditions.

## CONCLUSIONS

Our high-resolution analysis of the essentiality of all genic and intergenic loci in the *V. cholerae* genome provides a wealth of data that will be of considerable value for future studies of this important human pathogen. For example, knowledge of essential genes and pathways can inform screens for new drugs to treat cholera and facilitate an enhanced understanding of the specific metabolic and physiologic growth requirements for the organism. In addition, fine scale mapping allowed the identification of ‘subdomains’ in genes as well as in intergenic regions that are required for optimal growth. These regions included not only protein domains, but promoters and UTR sequences as well. Furthermore, overlay of transcriptomic data on top of our high-density transposon insertion data set facilitated identification of small ncRNA species that appear to regulate growth in *V. cholerae*.

The approach we developed here for analysis of high-density transposon mutagenesis data should be broadly applicable and useful for future transposon-insertion sequencing studies. We propose that several factors should routinely be considered when analyzing transposon-insertion sequencing data. First, correction for DNA replication—especially if data sets from different growth conditions will be compared—allows direct comparisons of data sets without bias from the rate of replication in each experiment. Second, genomically unbiased analysis pipelines, such as the SW approach, will permit characterization of the requirement for unannotated loci (both within and outside of coding regions) in bacterial growth. Finally, and perhaps most importantly, incorporation of an HMM-based filter can enhance the biological signal over the intrinsic noise in high-density transposon data sets because the HHM takes advantage of a feature inherent in all transposon-insertion sequencing data sets—local context information—that has not been exploited in previous analyses. Additionally, with some modifications, an HMM approach will also be useful for nonmariner–based transposon insertion sequence techniques such as TraDIS (6). Moreover, HMM-based approaches should be adaptable for analyses of conditional essentiality, where data from multiple conditions and genetic backgrounds are compared. Thus, we anticipate that our study will be the first of many applications for HMM-based analyses of transposon-insertion sequencing data.

## SUPPLEMENTARY DATA

Supplementary Data are available at NAR Online.

Supplementary Data
