# Supplementary material for: High-resolution definition of the Vibrio cholerae essential gene set with hidden Markov model–based analyses of transposon-insertion sequencing data
Source: Nucleic Acids Res. 2013 Jul 30;41(19):9033–48. doi: 10.1093/nar/gkt654 (PMC3799429; doi:10.1093/nar/gkt654)
Supplement: Supplementary Data [file supp_41_19_9033__index.html]

High-resolution definition of the Vibrio cholerae essential gene set with hidden Markov model–based analyses of transposon-insertion sequencing data — High-resolution definition of the Vibrio cholerae essential gene set with hidden Markov model–based analyses of transposon-insertion sequencing data — Supplementary Data 

# High-resolution definition of the *Vibrio cholerae* essential gene set with hidden Markov model–based analyses of transposon-insertion sequencing data

## 

files

**Files in this Data Supplement:**

- Supplementary Data - pdf file
- Supplementary Data - xlsx file
- Supplementary Data - txt file
- Supplementary Data - zip file
