# Supplementary material for: High-resolution definition of the Vibrio cholerae essential gene set with hidden Markov model–based analyses of transposon-insertion sequencing data
Source: Nucleic Acids Res. 2013 Jul 30;41(19):9033–48. doi: 10.1093/nar/gkt654 (PMC3799429; doi:10.1093/nar/gkt654)
Supplement: Supplementary Data [file supp_gkt654_nar-01659-h-2013-File011.pdf]

Supplementary Figure Legends

Figure S1. Himar1 insertion site distribution in *V. cholerae*

(A) The distribution of the distances between adjacent potential Himar1 transposon insertion sites (TA dinucleotides) on each chromosome was plotted for all TA sites (black), sites within 'genic' annotated open reading frames (blue), and in intergenic regions (red).

(B) The number of transposon insertions per locus was determined for genic and intergenic loci, and the number of loci with each insertion frequency is plotted for each chromosome.

(C) The distribution of total reads per transposon insertion site was plotted for sites on each chromosome.

Figure S2. Riboflavin uptake and utilization in *V. cholerae*.

Two technical replicates of *ribE*::Tn (*vc2268::Tn*) cultures were grown in the presence or absence of riboflavin (B2). Culture density (OD600) is plotted as a function of time.

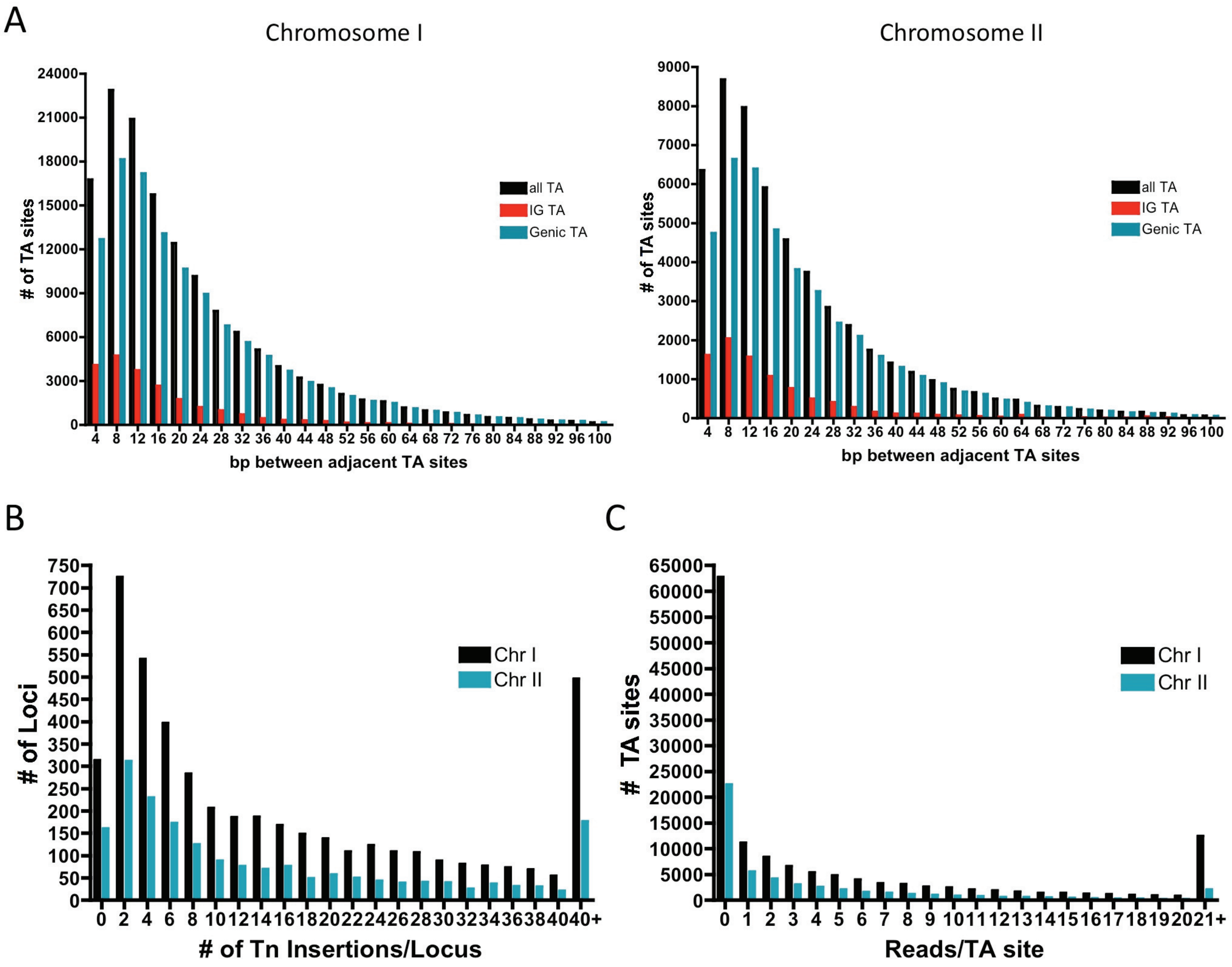
A
Chromosome I
# of TA sites
bp between adjacent TA sites
all TA
IG TA
Genic TA
Chromosome II
# of TA sites
bp between adjacent TA sites
all TA
IG TA
Genic TA
B
# of Loci
# of Tn Insertions/Locus
Chr I
Chr II
C
TA sites
Reads/TA site
Chr I
Chr II

Figure S1

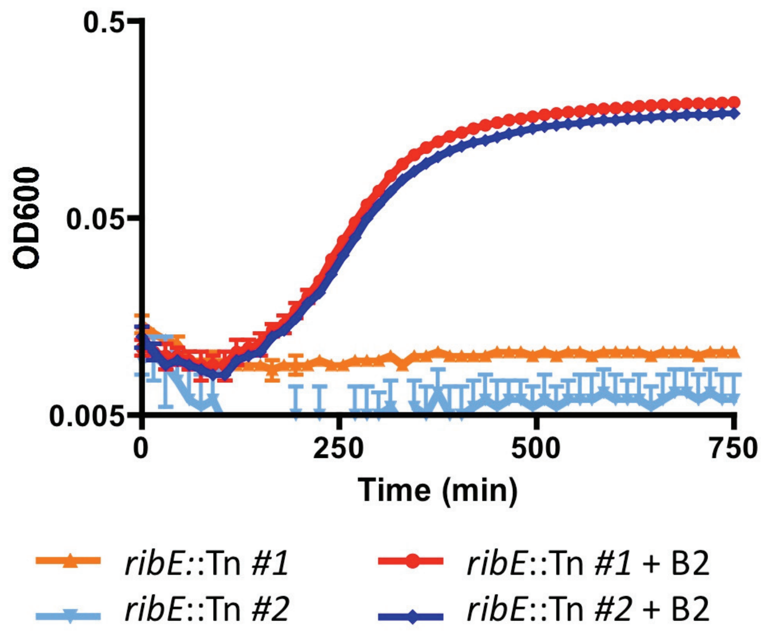
0.5
0.05
0.005
OD600
0
250
500
750
Time (min)
ribE::Tn #1
ribE::Tn #2
ribE::Tn #1 + B2
ribE::Tn #2 + B2

Figure S2
